# Knowledge, attitudes, and practices towards COVID-19 and associated factors in the mexican population

**DOI:** 10.15649/cuidarte.3565

**Published:** 2024-07-16

**Authors:** María del Pilar Ramírez-Díaz, Xunaxhi Guadalupe Núñez-Rasgado, Gabriel Hernández-Ramírez, Edna Isabel Rodríguez-López, Jorge Fernando Luna-Hernández

**Affiliations:** 1 Unidad de Ciencias Biológicas y de la Salud. Universidad del Istmo. Campus Juchitán, Oaxaca, México. E-mail: pilar.ramirezdiaz@gmail.com Universidad del Istmo Unidad de Ciencias Biológicas y de la Salud Universidad del Istmo Juchitán Oaxaca Mexico pilar.ramirezdiaz@gmail.com; 2 Unidad de Ciencias Biológicas y de la Salud. Universidad del Istmo. Campus Juchitán, Oaxaca, México. E-mail: xuny.gnr1398@gmail.com Universidad del Istmo Unidad de Ciencias Biológicas y de la Salud Universidad del Istmo Juchitán Oaxaca Mexico xuny.gnr1398@gmail.com; 3 Unidad de Ciencias Biológicas y de la Salud. Universidad del Istmo. Campus Juchitán, Oaxaca, México. E-mail: gabriel 25r@hotmail.com Universidad del Istmo Unidad de Ciencias Biológicas y de la Salud Universidad del Istmo Juchitán Oaxaca Mexico gabriel 25r@hotmail.com; 4 Unidad de Ciencias Biológicas y de la Salud. Universidad del Istmo. Campus Juchitán, Oaxaca, México. E-mail: edds.rdz@gmail.com Universidad del Istmo Unidad de Ciencias Biológicas y de la Salud Universidad del Istmo Juchitán Oaxaca Mexico edds.rdz@gmail.com; 5 Unidad de Ciencias Biológicas y de la Salud. Universidad del Istmo. Campus Juchitán, Oaxaca, México. E-mail: analistaver09@gmail.com Universidad del Istmo Unidad de Ciencias Biológicas y de la Salud Universidad del Istmo Juchitán Oaxaca Mexico analistaver09@gmail.com

**Keywords:** COVID-19, Knowledge, Attitude, Risk Factors, Mexico, COVID-19, Conocimiento, Actitud, Factores de Riesgo, México, COVID-19, Conhecimento, Atitude, Fatores de Risco, México

## Abstract

**Introduction:**

The COVID-19 disease has been one of the most harmful pandemics for humanity; therefore, ensuring adherence to preventive and control measures is essential. This adherence largely depends on the population's knowledge, attitudes, and practices (KAP) towards COVID-19.

**Objective:**

To identify the level of knowledge, attitudes, and practices towards COVID-19 prevention and its associated factors in Oaxaca, Mexico.

**Materials and Methods:**

This cross-sectional and analytical study surveyed adults online regarding KAP. Descriptive statistics and Chi-square or Fisher's exact tests were used for categorical comparisons. Factors associated with higher KAP levels were analyzed by calculating odd ratios (ORs) through logistic regression.

**Results:**

A total of 176 people participated, of whom 82.42% were women, and 81.83% were <30 years old. Among the participants, 90.88% identified direct contact with infected individuals as the main mode of transmission, 73.86% correctly identified the incubation period, and 93.22% referred to handwashing as the main preventive measure. The KAP scores were 65.34%, 32.95%, and 40.91%, respectively, with no differences by sex or age.

**Discussion:**

Participants with higher COVID-19 knowledge and more positive attitudes towards the pandemic were associated with more frequent preventive practices [OR:1.89 (CI:1.17- 3.73)] and [OR:3.21 (CI:1 .62-6.35)], respectively, compared to those with lower scores.

**Conclusions:**

The study population has a low level of KAP; greater knowledge about COVID-19 and more positive attitudes towards the pandemic increase preventive practices.

## Resumen

**Introducción:**

La enfermedad de COVID-19 ha sido una de las pandemias más perjudiciales para la humanidad, por lo cual, garantizar la adherencia a medidas preventivas y de control es fundamental. Esta adherencia depende en gran medida de los conocimientos, actitudes y prácticas (CAP) de la población frente al COVID-19.

**Objetivo:**

Identificar el nivel de conocimientos, actitudes y prácticas hacia la prevención de COVID-19 y sus factores asociados en Oaxaca, México.

**Materiales y Métodos:**

Estudio transversal y analítico, en mayores de edad a través de una encuesta en línea sobre CAP. Se aplicó estadística descriptiva, y chi cuadrada o exacta de Fisher para comparaciones categóricas. Se analizaron los factores asociados a un mayor nivel de CAP calculando OR's a través de regresión logística.

**Resultados:**

En total 176 personas participaron, de los cuales 82,42% fueron mujeres y 81,83% fueron <30 años. El 90,88% identificó como principal medio de transmisión el contacto directo con personas enfermas, 73,86% identificó correctamente el periodo de incubación y el 93,22% refirió el lavado de manos como principal medida preventiva. Las puntuaciones CAP fueron 65,34%, 32,95% y 40,91% respectivamente, sin diferencias por sexo y edad.

**Discusión:**

Los participantes con mayor conocimiento sobre COVID-19 y actitudes más positivas hacia la pandemia se asociaron con tener prácticas preventivas más frecuentes, [OR:1,89 (IC:1,17-3,73)] y [OR:3,21(IC:1,62-6,35)] respectivamente, en comparación con aquellos con menor puntuación.

**Conclusiones:**

La población de estudio tiene un bajo nivel de CAP. Un mayor conocimiento sobre COVID-19 y actitudes más positivas hacia la pandemia aumentan las prácticas preventivas.

## Resumo

**Introdução:**

A doença COVID-19 tem sido uma das pandemias mais prejudiciais para a humanidade, portanto, garantir a adesão às medidas preventivas e de controle é fundamental. Esta adesão depende em grande parte dos conhecimentos, atitudes e práticas (CAP) da população relativamente à COVID-19.

**Objetivo:**

Identificar o nível de conhecimento, atitudes e práticas em relação à prevenção da COVID-19 e seus fatores associados em Oaxaca, México.

**Materiais e Métodos:**

Estudo transversal e analítico, em adultos através de inquérito online sobre CAP. Estatísticas descritivas e qui-quadrado ou estatística exata de Fisher foram aplicadas para comparações categóricas. Os fatores associados ao maior nível de CAP foram analisados por meio do cálculo de OR por meio de regressão logística.

**Resultados:**

Participaram no total 176 pessoas, das quais 82,42% eram mulheres e 81,83% tinham <30 anos. 90,88% identificaram o contacto direto com pessoas doentes como principal meio de transmissão, 73,86% identificaram corretamente o período de incubação e 93,22% referiram a lavagem das mãos como principal medida preventiva. Os escores do CAP foram de 65,34%, 32,95% e 40,91% respectivamente, sem diferenças por sexo e idade.

**Discussão:**

Participantes com maior conhecimento sobre a COVID-19 e atitudes mais positivas frente à pandemia foram associados a práticas preventivas mais frequentes, [1,89 (IC:1,17-3,73)] e [3,21(IC:1,62-6,35)] respectivamente, comparados para aqueles com pontuação mais baixa.

**Conclusões:**

A população estudada apresenta baixo nível de CAP. Maior conhecimento sobre a COVID-19 e atitudes mais positivas frente à pandemia aumentam as práticas preventivas.

## Introduction

COVID-19 disease, caused by the SARS-CoV-2 virus, has been one of the most damaging pandemics for humanity and, according to the World Health Organization (WHO), 6,972,152 cumulative deaths had been reported globally as of October 2023^1^. Although the clinical manifestations of SARS-CoV-2 are not yet clearly understood, the main effects of the virus have been observed in the respiratory tract, with symptoms of varying severity^2^. The severity of COVID-19 has been associated with several risk factors, including advanced age, male sex, and underlying comorbidities such as some chronic diseases^3^.

Unfortunately, community spread of the virus continues in the country; therefore, ensuring successful disease control and adherence to preventive and control measures is critical. This adherence depends, to a large extent, on the population's knowledge, attitudes, and practices (KAP) regarding COVID-19, according to KAP theory^4^. It has been documented that public attitudes that promote compliance with government pandemic measures are significantly influenced by the level of knowledge about COVID-19^5^. In addition, preventive actions are essential in contexts where vaccination coverage has not been adequate or in populations whose beliefs prevent successful vaccination, as in the case of the anti-vaccine movement^6^. Several studies have reported that factors such as age, sex, educational level, socioeconomic level, occupation, and indigenous self identification, among others, can condition the level of knowledge that would lead to an impact on attitudes and practices regarding COVID-19, with great heterogeneity between countries and regions^7^. In addition, the KAP theory-based assessment of a target population allows for the structuring and implementation of more effective health education interventions tailored to a specific population or new situations, such as the one generated by the COVID-19 pandemic, enabling the adoption of beneficial behaviors to achieve a healthy lifestyle^8^.

Oaxaca is one of the poorest states in Mexico, with unique socioeconomic and cultural characteristics. Nearly 64% of the population report some level of poverty, approximately 30% of the total population consider themselves indigenous, and nearly 27% have a low level of education^9^. These characteristics have hindered the dissemination of information about COVID-19, which could affect the effectiveness of pandemic prevention measures, especially in communities with weak health information and services. However, no studies were found on KAP in the Oaxacan population that could provide a reference to the state of knowledge about the disease. Therefore, this study aimed to identify the level of knowledge, attitudes, and practices towards COVID-19 prevention and its associated factors in the population of Oaxaca.

## Materials and Methods

A cross-sectional observational study with an analytical component was conducted. The study universe consisted of people over 18 years with Internet access who live in one of the regions of the state of Oaxaca, Mexico. The study was conducted from August 8^th^ to September 1^st^, 2021.

### Sample

The sample was calculated taking into account the proportion expected according to the level of knowledge about the importance of "handwashing" (80.36%) reported by Vleitez et al.^10^ The population with Internet access in Oaxaca (445,212), according to the 2020 National Survey on the Availability and Use of Information Technologies in Households (ENDUTIH, for its acronym in Spanish)^11^, was also taken into account. A final sample of 246 participants was obtained using non- probabilistic snowball sampling. The study included individuals over 18 with an Internet connection who signed the digital informed consent form. Participants who did not understand Spanish or could not read were excluded, and all incomplete surveys were discarded.

### Data collection

A semi-structured, self-administered questionnaire was designed online through the Google Forms platform. It included questions on sociodemographic aspects, the general perception of COVID-19, the mode of transmission, the incubation period, symptoms, risk factors, and prevention initiatives. In addition, the questionnaire included a section on COVID-19 knowledge, attitudes, and practices, as well as questions on the influence of diet and nutritional status during the development of COVID-19.

The KAP survey was validated and adapted from previously published literature on viral epidemics related to Middle East Respiratory Syndrome (MERS-CoV)^12^. The original survey consists of 19 questions (6 for knowledge, 6 for attitude, and 7 for practice) with an overall Cronbach's alpha of 0.73, indicating acceptable internal consistency^12^. For this study, two questions on vaccination were added to the attitude dimension, bringing the total number of questions in this dimension to 8. In the sample analyzed, the instrument showed an overall Cronbach's alpha of 0.68, lower than the original survey and at the limit of what is acceptable for this type of instrument^12^. The dimensions of the instrument are rated as follows:

- Knowledge: (Cronbach's alpha of 0.71), 6 questions [yes= 1 point, no= 0 points, and I don't know= 0 points] with a total score of 6 points. Scores > 4 points were classified as “more accurate knowledge.”
- Attitudes: (Cronbach's alpha of 0.65), 8 questions [agree = 2 points, undecided= 1 point, and disagree= 0 points] for a total of 16 points. Scores > 15 points were classified as “more positive attitudes.”
- Practice: (Cronbach's alpha of 0.68), 7 questions [yes= 1 point, sometimes= 0 points, and I don't know= 0 points] for a total of 7 points. Scores > 6 points were classified as “most frequent practices.”

Finally, a section of questions was added on the population's perspective on the influence of nutritional status and chronic non-communicable diseases on the development of COVID-19.

### Data analysis

The information was analyzed using SPSS v. 27 software. Descriptive statistics with frequencies and proportions were used for sociodemographic characteristics. Survey sections were analyzed considering the total population and its classification by sex and age group using the Chi-square test or Fisher's exact test for proportional differences. An analysis was performed to identify factors associated with higher levels of KAP, calculating ORs with their respective 95% confidence intervals by logistic regression and adjusting for confounders. The Hosmer-Lemeshow test was used to test the goodness of fit (p>0.05). The original database was stored in Mendeley Data^13^.

### Ethical considerations

The present study was based on the Declaration of Helsinki and the guidelines from the General Health Law and the Regulations of the General Health Law on Health Research, which classified this research as low-risk research. In addition, informed consent was obtained from each participant.

## Results

A total of 176 participants were included in the study, of whom 82.42% were female, and 81.83% reported being under 30 years old. Additionally, 26.75% identified themselves as Indigenous, 30.77% of households had more than five members, and 88.12% had education beyond secondary level (high school, university, and postgraduate education). Social networks and the Internet were the main source of information on COVID-19 (Table 1).

**Table 1:** Sociodemographic characteristics of the study population

Variable	Total n=176 (%)
Sex	
Female	145 (82.42)
Male	31 (17.58)
Age	
<30 years	144 (81.83)
>30 years	32 (18.17)
Indigenous self-identification	
Yes	47 (26.75)
No	129 (73.25)
Income	
^a^ <5500	109 (67.92)
>5500	67 (38.08)
Socioeconomic status	
Lower-middle	161 (91.55)
High	15 (8.45)
Family members	
>5	54 (30.77)
<5	122 (69.23)
Occupation	
Farmers, traders, homemakers, non-government employees	48 (27.33)
Students, government employees, healthcare workers	128 (72.67)
Marital status	
With a partner	32 (18.28)
Without a partner	144 (81.72)
Educational level	
>secondary	155 (88.12)
<secondary	21 (11.88)
Media	
^*^ Internet	126 (71.64)
^┴^ Traditional	50 (28.36)

^*^ *Includes social media and the Internet in general.* ^┴^ *Includes television, radio, and megaphoning.* ^*a*^ *Mexican pesos.*

Regarding transmission mode, 90.88% of the population reported contact with an infected person as the primary mode of transmission. Additionally, 73.86% agreed that the incubation period is 2 to 14 days, and 97.65% identified fever, dry cough, and shortness of breath as the main symptoms. Furthermore, 91.55% of respondents recognized older adults as a risk group. In terms of treatment,

57.39% reported supportive treatment as the main approach, while 38.57% mentioned vaccination. The main preventive measures identified by respondents were washing their hands with soap and water (93.22%) and using masks (85.79%). In addition, 37.48% of the respondents said that the main problem in the family was not being able to leave the house. Women reported cleaning purchased products as an important measure to protect the family (p=0.002) and headache as a primary symptom (p=0.012). In terms of age groups, the <30-year age group reported contact with an infected person as an important mode of transmission compared to participants >30 years (p<0.001). This group also identified the incubation period of 2 to 14 days (p=0.012) and headache and diarrhea as symptoms of COVID-19. Within the risk groups, the <30-year age group identified individuals with chronic diseases compared to those >30 years old (p<0.001). The <30-year age group also had better knowledge of prevention measures such as avoiding contact with infected people (p<0.001), using masks (p<0.001), keeping social distance (p<0.001), self-quarantine (p=0.006), and strengthening healthcare (<0.001) (Table 2).

**Table 2:** Comparison of perceptions of COVID-19 regarding mode of transmission, incubation period, symptoms, risk factors, prevention initiatives, and challenges by sex and age group

Question	Total n=176	Male n=31	Female n=145	p^a^	<30 years n=144	>30 years n=32	p^a^
1. How is COVID-19 spread?							
Direct transmission during coughing	128(71.02)	25(80.63)	103(71.04)	0.275	101(70.11)	27(84.44)	0.102
Touching contaminated surfaces	105(59.68)	18(58.14)	87(60.03)	0.842	90(62.54)	15(46.91)	0.103
Contact with infected animals	12(6.76)	4(12.87)	8(5.49)	0.139	11(7.61)	1(3.11)	0.359
Through eating infected animal products (e.g., meat, milk)	4(2.31)	1(3.22)	3(2.14)	0.695	4(2.81)	0(0.00)	0.445b
Close contact with an infected person	160(90.88)	28(90.31)	132(91.02)	0.900	138(95.82)	22(68.82)	<0.001
2. Symptoms appear after which of the following periods?				0.972			0.012
2-5 days	39(22.14)	7(22.58)	32(22.06)		30(20.83)	9(28.12)	
2-14 days	130(73.86)	23(74.19)	107(73.79)		111(77.08)	19(59.37)	
Don’t know	7(4.00)	1(3.22)	6(4.13)		3(2.08)	4(12.50)	
3. What are the symptoms of COVID-19?							
Fever, dry cough, difficult breathing	172(97.65)	30(96.79)	142(97.91)	0.543b	142(98.61)	30(93.81)	0.095
Sore throat and stuffy nose	89(50.53)	12(38.66)	77(53.12)	0.146	65(45.11)	24(75.05)	0.002
Headache	119(67.58)	15(48.36)	104(71.71)	0.012	103(71.54)	16(50.05)	0.019
Diarrhea	83(47.16)	14(45.18)	69(47.62)	0.802	74(51.38)	9(28.09)	0.017
4. Who is most at risk of COVID-19 infection?							
Elderly people	161(91.48)	28(90.25)	133(91.71)	0.730b	134(93.11)	27(84.44)	0.112
Pregnant women	101(57.12)	19(61.27)	82(56.61)	0.692b	83(57.63)	18(56.32)	0.886
Children	68(38.51)	11(35.48)	57(39.32)	0.839b	57(39.67)	11(34.45)	0.584
Individuals with cancer, diabetes, respiratory diseases	150(85.19)	25(80.56)	125(86.23)	0.428	133(92.38)	17(53.17)	<0.001
Migrants from other parts of the world having COVID-19	13(7.36)	4(12.88)	9(6.22)	0.249^b^	12(8.28)	1(3.12)	0.467b
5. Which of the following describes COVID-19 treatment?				0.904			0.942
Supportive treatment	101(57.38)	17(54.83)	84(57.93)		83(57.63)	18(56.25)	
Vaccine	68(38.65)	13(41.93)	55(37.93)		55(38.19)	13(40.62)	
Don't know	7(3.97)	1(3.22)	6(4.13)		6(4.16)	1(3.12)	
6. What to do to prevent coronavirus?							
Wash your hands with water and soup	164(93.17)	28(90.31)	136(93.83)	0.446	135(93.81)	29(90.62)	0.526
Avoid touching your eyes and nose with your hands	145(82.35)	26(83.87)	119(82.11)	0.811	118(81.92)	27(84.43)	0.744
Avoid contact with infected people	126(71.59)	20(64.46)	106(73.14)	0.336	115(79.91)	11(34.42)	<0.001
Use masks	151(85.78)	24(77.45)	127(87.64)	0.141	132(91.72)	19(59.41)	<0.001
Maintain social distance	145(82.36)	25(80.67)	120(82.76)	0.779	133(92.41)	12(37.54)	<0.001
Maintain self-quarantine	65(36.88)	13(41.86)	52(35.87)	0.525	60(41.76)	5(15.61)	0.006
Quarantine the entire family members at home	34(19.27)	9(29.03)	25(17.23)	0.131	34(23.67)	0(0.00)	0.001b
Strengthen healthcare	82(46.55)	13(41.91)	69(47.64)	0.567	80(55.69)	2(6.33)	<0.001
Temporarily restrict outside visitors from entering the home	72(40.87)	14(45.22)	58(40.04)	0.596	61(42.44)	11(34.42)	0.406
7. Have you taken any initiative to protect your family?							
Arrange for handwashing with soap inside and outside the home	133(75.62)	20(64.51)	113(77.92)	0.115	115(79.91)	18(56.31)	0.005
Wash your hands with soap after touching pets	46(26.11)	7(22.66)	39(26.96)	0.690	38(26.44)	8(25.06)	0.872
Clean the products purchased at the supermarket	138(75.42)	18(58.18)	120(82.81)	0.002	116(80.66)	22(68.74)	0.142
Take off clothing and shoes before entering the house	69(39.26)	6(19.43)	63(43.41)	0.013	67(46.55)	2(6.32)	<0,001
8. Have you faced any problems to create awareness in your family about COVID-19?							
Negligence about the severity of the disease	47(26.71)	10(32.31)	37(25.55)	0.441	39(27.11)	8(25.03)	0.810
Reluctance to use masks	30(17.03)	7(22.62)	23(15.91)	0.367	23(16.04)	7(21.91)	0.422
Not being able to avoid going out	66(37.48)	12(38.71)	54(37.22)	0.878	55(38.26)	11(34.44)	0.686
Not facing the problem	27(15.34)	2(6.52)	25(17.24)	0.130	21(14.69)	6(18.88)	0.554

^*a*^ *Chi-square test,* ^*b*^ *Fisher’s exact test*

### Knowledge

Regarding the knowledge dimension, 98.29% know that COVID-19 is a dangerous disease, and no differences were observed by sex; however, those >30 years old believe that it only affects humans (p=0.041). Nevertheless, the final score reflected that only 65.34% of the sample had a more accurate knowledge (Table 3).

**Table 3:** Comparison of COVID-19 knowledge by sex and age group

Question	Total n=176	Male n=31	Female n=145	p^a^	<30 years n=144	>30 years n=32	p^a^
1.Is COVID-19 a dangerous disease?				0.722			0.712
Yes	173(98.29)	31(100.00)	142(97.93)		141(97.91)	32(100.00)	
No	2(1.13)	0(0.00)	2(1.37)		2(1.38)	0(0.00)	
I don’t know	1(0.56)	0(0.00)	1(0.68)		1(0.69)	0(0.00)	
2. Does it affect only humans?				0.254			0.041
Yes	108(61.36)	16(51.61)	92(63.44)		84(58.33)	24(75.00)	
No	48(27.27)	9(29.03)	39(26.89)		45(31.25)	3(9.38)	
I don’t know	20(11.36)	6(19.35)	14(9.65)		15(10.41)	5(15.63)	
							
3. Does it transmit from humans to animals?				0.327			0.194
Yes	30(17.04)	8(25.80)	22(15.17)		28(19.44)	2(6.25)	
No	95(53.97)	14(45.16)	81(55.86)		76(52.77)	19(59.38)	
I don’t know	51(28.97)	9(29.03)	42(28.96)		40(27.77)	11(34.38)	
4. Does it transmit from animals to humans?				0.992			0.325
Yes	45(25.56)	8(25.80)	37(25.51)		38(26.38)	7(21.88)	
No	84(47.72)	15(48.38)	69(47.58)		65(45.13)	19(59.38)	
I don’t know	47(26.70)	8(25.80)	39(26.89)		41(28.47)	6(18.75)	
5. Is it transmitted by animal products (e.g., milk, meat)?				0.863			0.458
Yes	18(10.22)	4(12.90)	14(9.65)		15(10.41)	3(9.38)	
No	129(73.29)	22(70.96)	107(73.79)		103(71.52)	26(81.25)	
I don’t know	29(16.47)	5(16.12)	24(16.55)		26(18.05)	3(9.38)	
6. Is it transmitted in well-cooked products?				0.885			0.483
Yes	1(0.56)	0(0.00)	1(0.68)		1(0.69)	0(0.00)	
No	154(87.50)	27(87.09)	127(87.58)		124(86.11)	30(93.75)	
I don’t know	21(11.93)	4(12.90)	17(11.72)		19(13.19)	2(6.25)	
Knowledge				0.915			0.204
More accurate	115(65.34)	20(64.51)	95(65.51)		91(63.19)	24(75.00)	
Less accurate	61(34.65)	11(35.48)	50(34.48)		53(36.80)	8(25.00)	

^*a*^ *Chi-square test*

### Attitudes

Among the participants, 91.47% believe that it is crucial to report a suspected case. All the participants agreed on the importance of wearing a mask in crowded places, and 98.86% believe that they should continue using it even after being vaccinated. Differences by age were observed, with participants aged <30 years old agreeing on the importance of reporting a suspected case compared to those aged >30 years (p<0.001). Differences were also observed in home treatment and the importance of health education. However, only 32.95% exhibited more positive attitudes (Table 4).

**Table 4:** Comparison of the dimension of attitudes towards COVID-19 by sex and age group

Question	Total n=176	Male n=31	Female n=145	p^a^	<30 years n=144	>30 years n=32	p^a^
1. It is crucial to report a suspected case to health authorities				0.227			<0.001
Agree	161(91.47)	29(93.54)	132(91.03)		136(94.44)	25(78.12)	
Disagree	6(3.40)	2(6.45)	4(2.75)		1(0.69)	5(15.62)	
Undecided	9(5.11)	0(0.00)	9(6.20)		7(4.86)	2(6.25)	
2. It is important to use a mask in crowded places				N/A			N/A
Agree	176(100.00)	31(100.00)	145(100.00)		144(100.00)	32(100.00)	
Disagree	0(0.00)	0(0.00)	0(0.00)		0(0.00)	0(0.00)	
Undecided	0(0.00)	0(0.00)	0(0.00)		0(0.00)	0(0.00)	
3. It is important to get vaccinated, regardless of the vaccine.				0.516			0.081
Agree	128(72.72)	24(77.41)	104(71.72)		108(75.00)	20(62.50)	
Disagree	19(10.79)	4(12.90)	15(10.34)		12(8.33)	7(21.87)	
Undecided	29(16.47)	3(9.67)	26(17.93)		24(16.66)	5(15.62)	
4. It is important to use a mask in a crowded place, even if we are already vaccinated.				0.322^b^			0.241
Agree	174(98.86)	30(96.77)	144(99.31)		143(99.30)	31(96.87)	
Disagree	0(0.00)	0(0.00)	0(0.00)		0(0.00)	0(0.00)	
Undecided	2(1.13)	1(3.22)	1(0.68)		1(0.69)	1(3.12)	
5. It is important to wash your hands and face after coming outside				0.511			0.503
Agree	174(98.86)	31(100.00)	143(98.62)		142(98.61)	32(100.00)	
Disagree	0(0.00)	0(0.00)	0(0.00)		0(0.00)	0(0.00)	
Undecided	2(1.13)	0(0.00)	2(1.37)		2(1.38)	0(0.00)	
6. COVID-19 is a preventable disease				0.166			0.231
Agree	145(82.38)	23(74.19)	122(84.13)		119(82.63)	26(81.25)	
Disagree	4(2.27)	2(6.45)	2(1.37)		2(1.38)	2(6.25)	
Undecided	27(15.34)	6(19.35)	21(14.48)		23(15.97)	4(12.50)	
7. It can be treated at home				0.782			0.027
Agree	98(55.68)	17(54.83)	81(55.86)		74(51.38)	24(75.00)	
Disagree	22(12.50)	5(16.12)	17(11.72)		18(12.50)	4(12.50)	
Undecided	56(31.81)	9(29.03)	47(32.41)		52(36.11)	4(12.50)	
8. Health education plays an important role in preventing COVID-19.				0.252			0.023
Agree	164(93.18)	31(100.00)	133(91.72)		137(95.13)	27(84.37)	
Disagree	2(1.13)	0(0.00)	2(1.37)		2(1.8)	0(0.00)	
Undecided	10(5.68)	0(0.00)	10(6.89)		5(3.47)	5(15.62)	
Attitudes				0.928			0.850
More positive	58(32.95)	10(32.26)	48(33.10)		47(32.63)	11(34.37)	
Less positive	118(67.04)	21(67.74)	97(66.89)		97(67.36)	21(65.62)	

^*a*^ *Chi-square test,* ^*b*^ *Fisher’s exact test*

### Practices

It was observed that the most common practices were handwashing (94.89%) and avoiding touching the face and eyes (80.11%). No differences were observed by sex or age group; however, the overall score of practices is considered low, as only 40.91% of the population had more frequent preventive practices against COVID-19 (Table 5).

**Table 5:** Comparison of the dimension of COVID-19 practices by sex and age group

Question	Total n=176	Male n=31	Female n=145	p^a^	<30 years n=144	>30 years n=32	p^a^
1. Do you use tissues when coughing or sneezing?				0.214			0.287
Yes	89(50.57)	19(61.29)	70(48.28)		69(47.92)	20(62.50)	
No	43(24.43)	8(25.81)	35(24.14)		38(26.39)	5(15.63)	
Sometimes	44(25.00)	4(12.90)	40(27.59)		37(25.69)	7(21.88)	
2. Do you wash your hands frequently using water and soap?				0.660			0.572
Yes	167(94.89)	29(93.55)	138(95.17)		136(94.44)	31(96.88)	
No	0(0.00)	0(0.00)	0(0.00)		0(0.00)	0(0.00)	
Sometimes	9(5.11)	2(6.45)	7(4.83)		8(5.56)	1(3.13)	
3. Do you avoid touching your face and eyes?				0.599			0.439
Yes	141(80.11)	23(74.19)	118(81.38)		114(79.17)	27(84.38)	
No	7(3.98)	2(6.45)	5(3.45)		5(3.47)	2(6.25)	
Sometimes	28(15.91)	6(19.35)	22(15.17)		25(17.36)	3(9.38)	
4. Do you maintain social distance (or home quarantine)?				0.620			0.465
Yes	135(76.70)	23(74.19)	112(77.24)		110(76.39)	25(78.13)	
No	10(5.68)	1(3.23)	9(6.21)		7(4.86)	3(9.38)	
Sometimes	31(17.61)	7(22.58)	24(16.55)		27(18.75)	4(12.50)	
5. Have you eaten healthy food since the onset of the outbreak?				0.122			0.643
Yes	109(61.93)	18(58.06)	91(62.76)		87(60.42)	22(68.75)	
No	17(9.66)	6(19.35)	11(7.59)		14(9.72)	3(9.38)	
Sometimes	50(28.41)	7(22.58)	43(29.66)		43(29.86)	7(21.88)	
6. Have you maintained a healthy lifestyle since the onset of the outbreak?				0.122			0.261
Yes	94(53.41)	15(48.39)	79(54.48)		73(50.69)	21(65.63)	
No	13(7.39)	5(16.13)	8(5.52)		12(8.33)	1(3.13)	
Sometimes	69(39.20)	11(35.48)	58(40.00)		59(40.97)	10(31.25)	
7. Do you comply with all government regulations regarding COVID-19?				0.083			0.388
Yes	126(71.59)	18(58.06)	108(74.48)		101(70.14)	25(78.13)	
No	7(3.98)	3(9.68)	4(2.76)		7(4.86)	0(0.00)	
Sometimes	43(24.43)	10(32.26)	33(22.76)		36(25.00)	7(21.88)	
Practices				0.498			0.248
More frequent	72(40.91)	11(35.48)	61(42.07)		56(38.89)	16(50.0)	
Less frequent	104(59.09)	20(64.52)	84(57.93)		88(61.11)	16(50.0)	

^*a*^ *Chi-square test*

### Association analysis

In the association analysis, greater knowledge and more positive attitudes were associated with more frequent preventive practices, with ORs of 1.89 (CI:1.17-3.73) and 3.21(CI:1.62-6.35)], respectively. No other variable showed a significant association (Table 6).

### Nutritional status and chronic diseases

Finally, regarding the influence of nutritional status and comorbidities on the development and severity of COVID-19, 95.50% of the population believes that nutrition is essential for preventing COVID-19. Additionally, comorbidities such as obesity, diabetes, and hypertension are seen as factors that can increase the severity of the disease.

**Table 6:** Factors associated with a higher level of KAP in the study population

	Total n=176 (%)	Knowledge				Attitudes				Practices			
More accurate	Less accurate	^p^	Adjusted OR* (95% CI)	More positive	Less positive	^p^	Adjusted ORǂ (95% CI)	More frequent	Less frequent	^p^	Adjusted ORǂ (95% CI)
ǂKnowledge													
More accurate	115(65.34)	N/A	N/A	N/A	N/A	39(67.24)	76(64.41)	0.781	0.90(0.45-1.80)	53(73.62)	62(59.62)	0.045	1.89(1.17-3.73)
Less accurate	61(34.66)	N/A	N/A	N/A	N/A	19(32.76)	42(35.59)		1	19(26.38)	42(40.38)		N/A
ǂAttitudes													
More positives	58(33.00)	N/A	N/A	N/A	N/A	N/A	N/A	N/A	N/A	34(47.23)	24(23.08)	0.001	3.21(1.62-6.35)
Less positives	118(67.00)	N/A	N/A	N/A	N/A	N/A	N/A	N/A	N/A	38(52.77)	80(76.92)		1
ǂPractices													
More frequent	72(40.90)	N/A	N/A	N/A	N/A	N/A	N/A	N/A	N/A	N/A	N/A	N/A	N/A
Less frequent	104(59.10)	N/A	N/A	N/A	N/A	N/A	N/A	N/A	N/A	N/A	N/A	N/A	N/A
^┴^Indigenous self-identification													
Yes	47(26.70)	30(26.08)	17(27.86)	0.478	1.33(0.60-2.93)	16(27.58)	31(26.27)	0.936	1.03(0.47-2.26)	20(27.77)	27(25.96)	0.944	0.97(0.44-2.13)
No	129(73.30)	85(73.92)	44(72.14)			42(72.42)	87(73.73)		1	52(72.23)	77(74.04)		1
^┴^Income													
^a^<5500	109(67.93)	68(59.13)	41(67.21)	0.112	1.83(0.86-3.89)	31(53.44)	78(66.10)	0.146	0.59(0.29-1.20)	44(61.11)	65(62.50)	0.619	1.20(0.58-2.47)
>5500	67(38.07)	47(40.86)	20(32.79)			27(46.56)	40(33.90)		1	28(38.89)	39(37.50)		1
^┴^Socioeconomic status													
Lower-middle	161(91.47)	48(41.73)	20(32.78)	0.108	1.85(0.87-3.93)	52(89.65)	109(92.37)	0.771	1.11(0.52-2.36)	64(88.88)	97(93.27)	0.426	0.73(0.35-1.55)
High	15(8.53)	67(58.26)	41(67.22)			6(10.35)	9(7.63)		1	8(11.12)	7(6.73)		1
^┴^Family members													
>5	54(30.68)	36(31.30)	18(29.50)	0.686	1.15(0.56-2.35)	14(24.13)	40(33.89)	0.202	0.61(0.29-1.29)	25(34.72)	29(27.88)	0.190	1.60(0.79-3.25)
<5	122(69.32)	79(68.69)	43(70.50)			44(75.87)	78(66.11)		1	47(65.23)	75(72.12)		1
^┴^Occupation													
Farmers, traders, homemakers, non-government employees	48(27.27)	36(31.30)	12(19.67)	0.220	1.76(0.71-4.38)	17(29.31)	31(26.27)	0.964	1.01(0.44-2.31)	23(31.94)	25(24.04)	0.721	1.16(0.51-2.63)
Students, government employees, healthcare workers	128(72.73)	79(68.69)	49(80.33)			41(70.69)	87(73.73)		1	49(68.06)	79(76.96)		1
^┴^Marital status													
With a partner	32(18.18)	21(18.26)	11(18.03)	0.351	0.61(0.22-1.69)	13(22.41)	19(16.11)	0.411	1.48(0.58-3.77)	17(23.61)	15(14.42)	0.395	1.51(0.58-3.89)
Without a partner	144(81.82)	94(81.79)	50(82.97)			45(77.59)	99(83.89)		1	55(76.39)	89(85.58)		1
Educational level													
>secondary	155(88.06)	100(86.95)	55(90.16)	0.435	1.81(0.40-8.09)	50(86.20)	105(88.98)	0.674	0.74(0.19-2.90)	61(84.72)	94(90.38)	0.882	0.90(0.23-3.47)
<secondary	21(11.94)	15(13.05)	6(9.84)			8(13.80)	13(11.02)		1	11(15.28)	10(9.62)		1
Media to receive information about COVID-19													
Internet: social media and the internet in general	58(33.00)	84(73.04)	42(68.85)	0.260	1.60(0.70-3.63)	43(34.12)	15(30.00)	0.424	1.43(0.59-3.50)	50(39.68)	22(44.00)	0.885	1.06(0.46-2.43)
^┴^Traditional: television, radio, and megaphoning	118(67.00)	31(26.95)	19(31.15)			83(65.88)	35(70.00)		1	76(60.32)	28(56.00)		1

^*a*^ *Mexican pesos,* ^ǂ^ *Adjusted for sex, age, Indigenous self-identification, income, number of family members, socioeconomic status, educational level, and marital status,* ^┴^ *Adjusted for sex and age. 1: Reference category.*

## Discussion

This study was conducted with the objective of identifying the level of KAP towards COVID-19 and its associated factors in the population of Oaxaca. Regarding COVID-19 perception, almost the entire population identified contact with an infected person and direct transmission during coughing as the primary means of infection. In terms of main symptoms, participants reported the presence of fever, dry cough, and difficulty breathing; handwashing was the most frequent preventive measure, similar to what has been reported in other studies^12^^, ^^14^^, ^^18^.

Regarding treatment, just over half of the study population reported that supportive treatment was the best alternative, and a third of the population considered vaccination as a treatment, which differs from what Ferdous et al^.(^^12^ reported in their research in Bangladesh, where only 1% of the population believed in vaccination as a treatment. This difference is interesting because the population studied in Bangladesh had higher educational and socioeconomic levels than the population studied in this study. However, it is important to note that the difference in time between one study and the other is one year, which would explain why the population of Bangladesh would not consider a vaccine as part of the treatment, as there was no such alternative in the early stages of the pandemic^11^. The groups identified at risk were mainly older adults and people with chronic or respiratory diseases; these results are similar to those reported in several studies in Lebanon^16^, Bangladesh^12^, Vietnam^19^, and Mexico^10^.

According to the KAP results, it was expected that women would have a higher level of knowledge, as it has been reported that women make greater use of health services and have better handwashing habits than men, a key practice against COVID-19^21^^, ^^22^. However, no sex differences were observed in our study, in contrast to what has been reported in other studies^15^^, ^^19^^, ^^23^ where women showed having greater knowledge. In contrast, in a study by Amalakanti et al. in India, women had less knowledge and preventive practice of COVID-19 than men^24^, perhaps due to cultural and social issues, as women in that country have more social restrictions on education and health services. In addition, the results of this research are similar to those of a systematic review on KAP, which found no sex differences in COVID-19 knowledge, attitudes, and practices^7^. In this sense, we could think that studies of knowledge from a gender perspective are influenced by geographical, social, and cultural factors. Overall, two- thirds of the population showed more accurate knowledge, similar to what has been reported in other developing countries such as Vietnam^19^.

In terms of attitudes, it was observed that those under 30 have better attitudes because they believe that reporting a suspected case is important. Regarding the importance of health education, these results contradict those reported by Demba et al. in Guinea, where it was observed that participants under 30 years of age had more negative attitudes, which could be explained by the fact that the studied population was mostly students and had a higher level of education, greater access to information and, possibly because of this, more positive attitudes. This was also found in another study by Ramos et al. in Ecuador^26^, where participants with a bachelor's or master's degree were more optimistic about the pandemic than those with less education. Notably, only one-third of the population had a more positive attitude, half the number reported by Ferdous et al.^12^ in Bangladesh but almost twice the number reported in Guinea^25^.

In terms of preventive practices, handwashing and avoiding touching the face were the most frequent, similar to that reported by Al-Hussami et al. in the Jordanian population. In general, half of the population practiced more frequent care, a lower proportion than that reported in Asian and African countries such as Bangladesh, Nigeria, and Iran^11^^, ^^28^^, ^^29^.

In the association analysis, greater knowledge increases the likelihood of more frequent preventive practices, consistent with the results of a systematic review and meta-analysis of KAP and COVID-19^25^. In this sense, a more positive attitude towards the pandemic increased the probability of having more frequent protective practices threefold. Therefore, having good knowledge and a positive attitude towards the health situation improves preventive practices against COVID-19. It is worth noting that almost all respondents reported knowing that diet and proper nutrition can reduce complications from COVID-19 as well as the likelihood of getting sick. However, only half of the population has considered maintaining a healthy lifestyle since the onset of the pandemic, according to other research conducted in Latin America^27^.

In general, the study population had a low KAP score despite participants' good level of education. However, it must be considered that the state of Oaxaca has a high cultural and traditional load that can condition the inhabitants' behavior. For example, one-third of the study population self-identified as Indigenous, and it is possible that due to cultural, religious, and traditional influences, they may not follow some recommendations, such as avoiding gatherings, festivities, and crowds.

The limitations of this research lie in the nature of the study, as the instrument used was an online survey, which prevented the achievement of the calculated representative sample. It is also important to note that some KAP surveys conducted at the beginning of the pandemic differ from the updated surveys, making it difficult to compare results with other research. Despite these limitations, there is little information on COVID-19 KAP at the state and national levels, which is why this study is positioned as a pioneer in the field and a reference for other research in the state of Oaxaca.

## Conclusion

The study population has a low level of KAP towards COVID-19. Greater knowledge and more positive attitudes increased the likelihood of more frequent preventive practices. The findings should be instrumental in planning health education programs, especially for promoting preventive behaviors, since sound knowledge, positive attitudes, and correct practices can make all the difference in the battle against COVID-19.
